# Impact of Terrestrial Input on Deep-Sea Benthic Archaeal Community Structure in South China Sea Sediments

**DOI:** 10.3389/fmicb.2020.572017

**Published:** 2020-11-05

**Authors:** Dengxun Lai, Brian P. Hedlund, Wei Xie, Jingjing Liu, Tommy J. Phelps, Chuanlun Zhang, Peng Wang

**Affiliations:** ^1^State Key Laboratory of Marine Geology, Tongji University, Shanghai, China; ^2^School of Life Sciences, University of Nevada, Las Vegas, NV, United States; ^3^Nevada Institute of Personalized Medicine, University of Nevada, Las Vegas, NV, United States; ^4^School of Marine Sciences, Sun Yat-sen University, Zhuhai, China; ^5^Southern Marine Science and Engineering Guangdong Laboratory (Zhuhai), Zhuhai, China; ^6^Earth and Planetary Sciences, University of Tennessee, Knoxville, Knoxville, TN, United States; ^7^Shenzhen Key Laboratory of Marine Archaea Geo-Omics, Southern University of Science and Technology, Shenzhen, China; ^8^Department of Ocean Science and Engineering, Southern University of Science and Technology, Shenzhen, China; ^9^Southern Marine Science and Engineering Guangdong Laboratory (Guangzhou), Guangzhou, China; ^10^Shanghai Sheshan National Geophysical Observatory, Shanghai, China

**Keywords:** benthic archaea, *thermoprofundales*, *thaumarchaeota*, terrestrial input of organic matter, glacial-interglacial cycles

## Abstract

Archaea are widespread in marine sediments and play important roles in the cycling of sedimentary organic carbon. However, factors controlling the distribution of archaea in marine sediments are not well understood. Here we investigated benthic archaeal communities over glacial-interglacial cycles in the northern South China Sea and evaluated their responses to sediment organic matter sources and inter-species interactions. Archaea in sediments deposited during the interglacial period Marine Isotope Stage (MIS) 1 (Holocene) were significantly different from those in sediments deposited in MIS 2 and MIS 3 of the Last Glacial Period when terrestrial input to the South China Sea was enhanced based on analysis of the long-chain n-alkane C_31_. The absolute archaeal 16S rRNA gene abundance in subsurface sediments was highest in MIS 2, coincident with high sedimentation rates and high concentrations of total organic carbon. Soil Crenarchaeotic Group (SCG; *Nitrososphaerales*) species, the most abundant ammonia-oxidizing archaea in soils, increased dramatically during MIS 2, likely reflecting transport of terrestrial archaea during glacial periods with high sedimentation rates. Co-occurrence network analyses indicated significant association of SCG archaea with benthic deep-sea microbes such as *Bathyarchaeota* and *Thermoprofundales* in MIS 2 and MIS 3, suggesting potential interactions among these archaeal groups. Meanwhile, *Thermoprofundales* abundance was positively correlated with total organic carbon (TOC), along with n-alkane C_31_ and sedimentation rate, indicating that *Thermoprofundales* may be particularly important in processing of organic carbon in deep-sea sediments. Collectively, these results demonstrate that the composition of heterotrophic benthic archaea in the South China Sea may be influenced by terrestrial organic input in tune with glacial-interglacial cycles, suggesting a plausible link between global climate change and microbial population dynamics in deep-sea marine sediments.

## Introduction

Long-term carbon sequestration in the form of organic matter (OM) deposited in marine sediments plays an important role in climate regulation. Currently, an estimated 7.8 × 10^22^ grams of carbon are stored in marine sediments, including both terrestrial and marine sources (Mackenzie et al., 2004). The contributions of these distinct organic pools to marine sediment vary with climate, geologic time, and geographic location (Stein, 1990; Schubert and Calvert, 2001; Yamamoto and Polyak, 2009). Marine OM from organisms in the water column contains predominantly proteins and carbohydrates, whereas terrestrial components such as lignocellulose derived from vascular plants are generally more nitrogen-depleted (Hedges et al., 1997). It has been observed that marine OM is broadly more reactive than terrestrial OM (Prahl et al., 1997; Aller and Blair, 2004; Zhang et al., 2018; He et al., 2020) and thus remineralization of terrestrial OM is much less efficient than marine OM.

The differences in quantity and quality of organic matter likely have important influences on deep-sea microbial assemblages, which are generally considered food (organic carbon) limited (Smith et al., 2008). Bathyal sediments below 2000 m water depth comprise the majority of the sea floor (Dunne et al., 2007) and are generally oligotrophic, with low organic carbon content and low sedimentation rates (Seiter et al., 2004; Dunne et al., 2007). Due to low respiration rates, dissolved electron acceptors such as oxygen, nitrate, and sulfate can penetrate deep into oligotrophic sediments on the scale of meters (D’Hondt et al., 2004; D’Hondt et al., 2015). In contrast, coastal sediments are commonly rich in organic matter, which can consume those electron acceptors within millimeters to centimeters of the sediment depth.

Archaea, as an important component in the sedimentary biosphere, constitute a significant portion of the global biomass (Whitman et al., 1998; Danovaro et al., 2015; Hoshino and Inagaki, 2019) and are key drivers of organic matter remineralization in sediments (Biddle et al., 2006; Lloyd et al., 2013; Zhou et al., 2018). Genomic and transcriptomic data have shown that marine archaea can use diverse organic compounds including fatty acids, carbohydrates, and lipids as sources of carbon (Inagaki et al., 2006; Li et al., 2015). Archaea have been reported to be of similar abundance with bacteria and can even dominate in some marine sediments (Biddle et al., 2006; Lipp et al., 2008; Vuillemin et al., 2019). Some archaeal lineages are considered to constitute the *in situ* populations in typical subsurface sediments (Inagaki et al., 2006; Parkes et al., 2014). For example, *Lokiarchaeota* [previously referred to as Marine Benthic Group B (MBGB) or Deep-sea Archaeal Group (DSAG)] has been prominently observed in methane hydrate-bearing sediments (Inagaki et al., 2006; Nunoura et al., 2008; Dang et al., 2010). *Bathyarchaeota* [previously named Miscellaneous Crenarchaeotic Group (MCG)] is widespread in marine sediments, particularly the organic-rich sediments on continental margins (Kubo et al., 2012; Wang et al., 2020a). In addition, *Thermoprofundales* [formerly named Marine Benthic Group D (MBGD)], *Hadesarchaeota* [formerly named South African Gold Mine Euryarchaeotal Group (SAGMEG)], and *Halobacteriales* (*Halobacteriaceae*, Deep Sea Euryarchaeotic Group, and Marine Hydrothermal Vent Group) have also been found in various oceanic regions (Sørensen and Teske, 2006; Heijs et al., 2008; Wang et al., 2014; Wang et al., 2020b).

Ammonia-oxidizing archaea (AOA) of the phylum *Thaumarchaeota* are ecologically widespread, occurring in terrestrial and marine habitats worldwide, and significantly contribute to global nitrogen cycle (Alves et al., 2018). AOA are regarded as important players in fueling microbial communities and sustaining oxic deep-sea benthic ecosystems (Orsi, 2018). Marine group I (MG-I; also named *Nitrosopumilales* or Group I.1a), the major AOA group in marine environments, constitutes the most abundant archaeal group in the global oceans (Könneke et al., 2005; Qin et al., 2014, 2020). MG-I in marine sediments were found to be diverse and phylogenetically distinct from those in water columns (Francis et al., 2005; Durbin and Teske, 2010; Wang et al., 2010). In contrast, Soil Crenarchaeotic Group (SCG; also named *Nitrososphaerales* or Group I.1b) has been found in various terrestrial environments such as soils (Tourna et al., 2011; Bouskill et al., 2012; Hong and Cho, 2015; Schneider et al., 2015), freshwater aquatic systems (Liu et al., 2014; Fillol et al., 2015), and hot springs (Zhang et al., 2008). Recent research has reported the identification of SCG over a wide spectrum of marine sediments (Li et al., 2008; Park et al., 2008; Dai et al., 2016; Alves et al., 2018) and even in a hadal trench (Nunoura et al., 2013); thus, the occurrence and ecological significance of this lineage is not fully understood.

While it is recognized that archaea are ubiquitous and play a crucial role in elemental cycling (D’Hondt et al., 2002; Offre et al., 2013), our understanding of the functions of marine benthic archaea, and the influence of climatic forces on the composition and function of marine benthic communities is far from complete. The influence of glacial runoff on bacterial communities in fjord sediments has been suggested (Pelikan et al., 2019). Consistent with this observation, terrestrially derived sediments have been shown to retain indigenous bacterial communities in marine deep-sea sediments for tens of millions of years (Inagaki et al., 2015). Additionally, analysis of 16S rRNA gene clone libraries from subsurface continental slope sediments has revealed that differences in bacterial and archaeal community composition could be associated with depositional environments (Nunoura et al., 2009). However, studies are lacking that integrate biological analyses with terrestrial input biomarkers and the geological time frames or climatic processes.

The South China Sea is one of the largest margin seas. Organic matter on the continental slope has complex compositions, which are affected by climate oscillations, especially glacial and interglacial cycles (Zhao et al., 2017). The large expansion of exposed continental shelf as the sea level decreased during the Last Glacial Period, especially the Last Glacial Maximum (LGM), led to an expansion of vegetation around the northern South China Sea (Zhou et al., 2012). During that time, a large amount of terrigenous material, including terrestrially derived organic carbon (TerrOC), was transported to the deep ocean via river and submarine canyons (Lin et al., 2016).

In this study, archaeal 16S rRNA gene characterization was performed in sediment from the Pearl River Submarine Canyon in the South China Sea to identify and quantify archaeal species in the context of geochemical and paleoecological signatures, with a goal to understand the potential impact of terrestrial input on deep-sea benthic archaeal community structure in South China Sea sediments.

## Materials and Methods

### Geological Setting and Sample Collection

**FIGURE 1:**
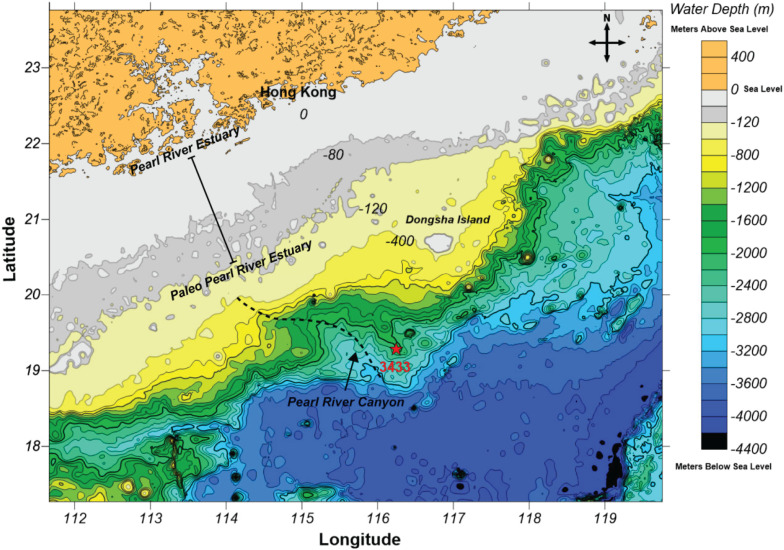
Geomorphologic map of the northern continental margin of the South China Sea. Color bar on the right side represents water depth. The sediment core location is indicated by the red star. The black dotted line is the Pearl River Canyon. The range of Pearl River Estuary is shown by a black solid line.

A sediment core from the Pearl River Submarine Canyon (MD12-3433Cq; 19°16.88′ N, 116°14.52′ E; Water depth: 2125 m) in the South China Sea was obtained (Figure 1) using a gravity corer during the Chinese-French joint MD190-CIRCEA cruise (June 12 to June 30, 2012). This site is located about 380 km southeast of Hong Kong. The core consisted of highly stratified sediments with colors from light (0 to 100 cmbsf) to deep gray (100 to 820 cmbsf). The sediments were characterized by continuous homogenous clay or silt without observable bioturbations, suggesting a stable depositional environment.

**FIGURE 2:**
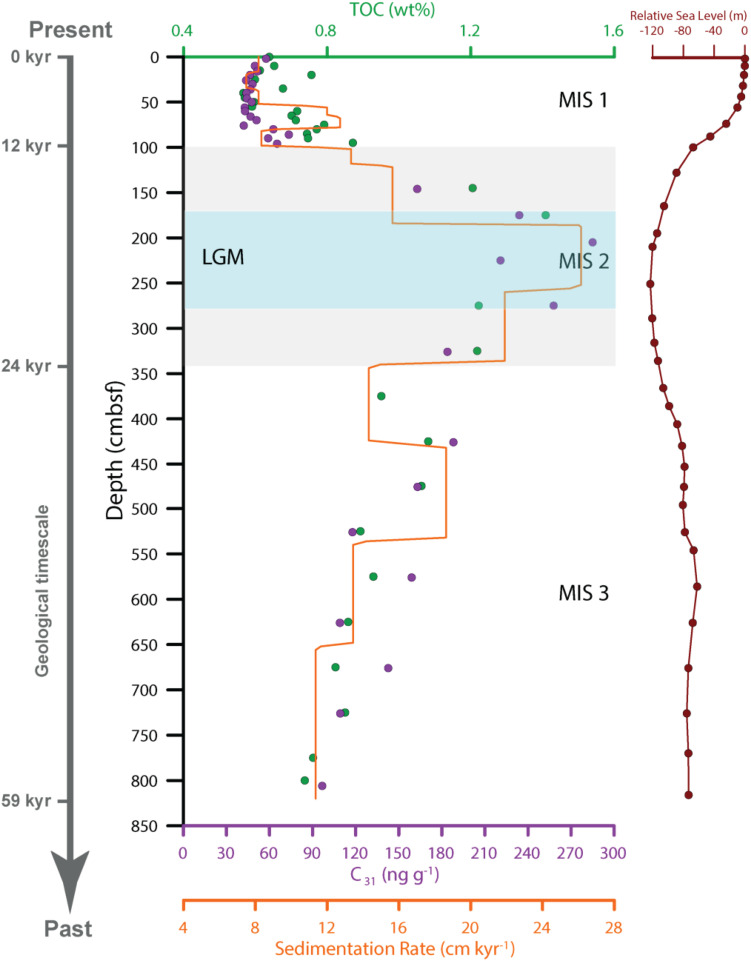
The depth profiles of total organic carbon (TOC), n-alkane C_31_, and sedimentation rate. The geological timescale (59 kyr-present) is shown on the right. The Relative Seal Level is from Higginson et al. (2003). Samples were assigned to MIS 1, MIS 2, and MIS 3 according to the age model established by (Zhao et al., 2017). LGM, Last Glacial Maximum.

On board the research vessel, samples for microbial (archaeal and bacterial) analyses were carefully taken from the center of the core using sterilized tools. Thirty-six subsamples were collected into airtight sterile PVC tubes and stored in a −80°C freezer until further analysis. Samples from the sediment core were assigned to one of the three Marine Isotope Stages (MISs) (Figure 2). Sediments in the top 1 m belonged to MIS 1, those of the 1–3.5 m depth MIS 2, and those below 3.5 m depth MIS 3. MIS 1 and MIS 3 represent interglacial periods while MIS 2 represents a glacial period. MIS 3 is interstadial during the Last Glacial Period, which spans from MIS 2 to MIS 4.

### Determination of Environmental Parameters

Total organic carbon (TOC) and total nitrogen were measured by Vario Cube CN (Germany Elementar Company). Briefly, sediment samples (around 0.5 g) were decarbonated by acidification with 1 N HCl, rinsed three times with deionized water until the pH value decreased to around 7, and dried using a freeze dryer. Finally, the dried samples (∼20 mg per sample) were manually ground, sealed in tin foil, and loaded onto the analyzer for analysis. In a similar manner, TC was measured without HCl treatment. In order to eliminate the deviation from the weight loss of carbonates, TOC was corrected using the following formula: TOC (%) = TOC_measured_ × (12−TC)/(12−TOC_measured_). Alkanes were measured by GC-MS (Agilent) at State Key Laboratory of Marine Geology (Tongji University) and grain size was measured using Laser Diffraction Particle Size Analyzer (Beckman Coulter LS230, United States). Water content was calculated as follows: water content = (wet weight – dry weight)/wet weight. The age model for this core was established by the planktonic foraminiferal (*Globigerinoides ruber*) δ^18^O curve (Zhao et al., 2017). Sedimentation rate (SR, unit:cm kyr^–1^) was estimated by age and the thickness of the sediment column.

Porewater samples were collected on board the ship from sediments using rhizon samplers (Seeberg-Elverfeldt et al., 2005), which were equipped with vacuum syringes. Approximately 5-10 ml volume of pore water was removed from the sediment and filtered with 0.22 μm membrane. A total of 28 samples were collected and frozen immediately at −20°C for storage. Nutrient analyses including nitrate, nitrite, ammonium and phosphate were performed on a continuous flow analyzer (AA3, Seal Analytical, Norderstedt, Germany). Sulfate and chloride contents were measured by ion chromatography (ICS-1500; Dionex, CA, United States) at Tongji University (Shanghai, China).

### DNA Extraction and 16S rRNA Gene Sequencing

Bulk DNA was extracted from approximately 0.5 g sediment of each sampling depth with the FastDNA^®^ spin kit for soil (MP Biomedicals, United States) following a modified version of the manufacturer’s protocol. According to the results of a pre-experiment, guanidinium thiocyanate (GTC) wash buffer (MP Biomedicals, United States) was used to improve the efficiency of DNA extraction. Sterilized quartz sand was used as control in each DNA extraction process. In total, 35 samples were included in the extraction. Archaeal 16S rRNA gene fragments were amplified using specific primers Arch_524F 5′-barcode-TGY CAG CCG CCG CGG TAA-3′ and 958_R 5′-YCC GGC GTT GAV TCC AAT T-3′ (Liu et al., 2016; Liu C. et al., 2017; Cerqueira et al., 2017), which cover the V4-5 regions. The V3 and V4 regions of the bacterial 16S rRNA genes were amplified using the bacterial universal primers 338F: 5′-ACTCCTACGGGAGGCAGCAG-3′ and 806R: 5′-GGACTACHVGGGTWTCTAAT-3′ (Fan et al., 2019). PCR reactions were conducted in triplicate in a 20 μL reaction volume containing 0.8 μl of each primer (5 μM), 4 μl of 5 × FastPfu Buffer, 0.4 μl of FastPfu Polymerase, 2 μl of 2.5 mM dNTPs, and 10 ng of template DNA. The PCR products were sent to Majorbio Bio-Pharm Technology Co., Ltd. (Shanghai, China) for sequencing.

Briefly, 16S rRNA gene amplicons were extracted from 2% agarose gels and purified using the AxyPrep DNA Gel Extraction Kit (Axygen Biosciences, Union City, CA, United States) according to the manufacturer’s instructions and quantified using QuantiFluor^TM^-ST (Promega, United States). The sequencing library was prepared using the NEXTFLEX^TM^ Rapid DNA-Seq Kit (BIOO Scientific Crop., Austin, TX, United States) following the manufacture’s recommendations. The purified amplicons were pooled and paired-end (PE) sequenced (2 × 250) on an Illumina MiSeq platform (Illumina Inc., San Diego, CA, United States) according to standard protocols. The raw reads were deposited into the NCBI Sequence Read Archive (SRA) (PRJNA563932 and PRJNA667744).

### Real-Time Quantitative PCR

Archaeal 16S rRNA genes were quantified by real-time quantitative PCR (PIKO REAL 96, Thermo Fisher Scientific, place, country) with Archaea-specific primers (Arch344F 5′-ACG GGG YGC AGC AGG CGC GA-3′ and Arch915R 5′-GTG CTC CCC CGC CAA TTC CT-3′) (Casamayor et al., 2002; Ohene-Adjei et al., 2007) targeting the V3-V5 region of the 16S rRNA gene. Each 10 μl qPCR reaction solution consisted of 5 μl SYBR Premix Ex TaqTM II (TaKaRa Bio Co., Kutsatsu, Japan), 1 μl of template DNA, 0.2 μl of 1 μM each primer, 0.1 μl Bovine Serum Albumin (BSA, 20 mg/mL) solution (TaKaRa Bio Co., Kutsatsu, Japan) and 3.5 μl deionized water. Sterilized water was used as a negative control. The procedures for qPCR were as follows: 95°C for 30 s; 35 cycles at 95°C for 5 s, 55°C for 30 s, and 72°C for 1 min. The qPCR was done in triplicates for each sample. The standard curve was obtained by using five 10-fold serial dilutions of purified plasmid DNA from the cloned archaeal 16S rRNA gene of *Nitrosopumilus maritimus* with the primer pair Arch21F and Arch958R (DeLong, 1992). The *R*^2^ values for the standard curves were greater than 0.98 and the amplification efficiencies were between 85 and 88%. qPCR results were rejected if the post-amplification melt curves showed evidence of primer dimers.

### Data Processing and Statistical Analysis

Raw Illumina fastq files were de-multiplexed, quality-filtered and analyzed using QIIME (Quantitative Insights into Microbial Ecology, version 1.9.1). Chimeric reads were filtered using UCHIME (Edgar et al., 2011) and Operational Taxonomic Units (OTUs) at 97% sequence similarity was done using UPARSE (version 7.1^1^). The taxonomy of each OTU was assigned using the RDP Classifier^2^ against the Silva (SSU123) database using a confidence threshold of 70%. All samples were normalized to the lowest number of sequences (19,195 reads), since uneven sequencing depth could affect microbial diversity estimates. Rarefaction curves indicated that sufficient reads were obtained for robust statistical analysis (Supplementary Figure 1).

Rarefaction curves, richness estimators (Chao) and diversity estimators (Shannon index) were calculated with Mothur software (version 1.34.4) (Schloss et al., 2009). Non-metric multidimensional scaling analysis (NMDS) was used to determine the degree of dissimilarity between pairs of archaeal communities using the Bray-Curtis distance method. NMDS and one-way analysis of similarity (ANOSIM: permutations = 999) were executed with the vegan package in R version 3.5.1. Visualization was handled in R using the ggplot2 graphics package (Wickham, 2011).

An estimate of the absolute abundance of 16S rRNA gene copies of SCG, MG-I, and *Thermoprofundales* per gram of sediment was calculated by multiplying the absolute archaeal 16S rRNA gene abundance (obtained by qPCR) by their respective relative abundance from 16S rRNA gene sequencing (Lou et al., 2018; Elovitz et al., 2019; Dorsaz et al., 2020).

Redundancy analysis (RDA) was conducted using PRIMER v6.1.16 (Plymouth Routines In Multivariate Ecological Research, PRIMER-E. Ltd., New Zealand) to examine the relationships between archaeal communities and environmental parameters. The distance matrix of archaeal groups, as response variables, was calculated using the Bray-Curtis method. A stepwise method with *R*^2^ value was applied for the selection of environmental factors. The statistical significance of the RDA was further tested using the Monte Carlo permutation test (999 permutations).

To examine associations between archaeal taxa, we analyzed pairwise correlations of the relative abundance of archaeal OTUs using Extended Local Similarity Analysis (eLSA) (Xia et al., 2011). OTU abundance data were filtered including a mean minimum occurrence of 2 OTUs per sample and the ratio of empty less than 1/3. *P*-values were estimated using the “perm” approach and “percentile Z” in the setting was used to normalize data (Xia et al., 2013). False discovery rates (FDR *Q*-values) were calculated to estimate the likelihood of false positives (Storey, 2003). The whole sediment column was separated into two parts: samples in MIS 1 and samples in MIS 2 and MIS 3, based on their similarity. OTUs with significant correlations (*P* ≤ 0.05 and *Q* ≤ 0.01) were visualized in Cytoscape (v3.7.0) (Shannon et al., 2003) together with an attribute table of taxonomy.

A phylogenetic tree of AOA based on the 16S rRNA gene was inferred with QuickTree (Howe et al., 2002) using the neighbor-joining method with 1000 bootstrap replicates. Numbers at branch nodes refer to bootstrap values. Sequences used for creating the phylogenetic tree include AOA sequences from MD12-3433, the corresponding best Blast hits from NCBI, published MG-I subgroup sequences (Durbin and Teske, 2010) and MG-I sequences from the water column of the South China Sea (Liu H. et al., 2017). Euryarchaeota sequences were used as an outgroup. Visualization and annotation were conducted in R software (V.3.5.1).

## Results

### Pore Water Chemistry

Chloride and sulfate were measured immediately after the pore water samples arrived at the home laboratory (Tongji University). Nitrate, nitrite, ammonium, and phosphate were measured in a later time at Tongji University. These parameters tended to be unstable during measurements and the pore water samples were transferred between two different labs, and may not have been preserved appropriately during storage. Thus, only chloride and sulfate were reported in this study (Supplementary Figure 2).

### Sedimentation Rate, TOC and n-Alkane C_31_

The sedimentation rate varied between 11.4 and 18.6 cm kyr^–1^ during MIS 3 and showed a general increasing trend with younger sediments. On average, the sedimentation rates were 14.0 ± 2.7 cm kyr^–1^ in MIS 3, 19.6 ± 4.8 cm kyr^–1^ in MIS 2, and 9.2 ± 1.9 cm kyr^–1^ in MIS 1 (Figure 2). The sedimentation rates in the three MISs differed significantly as determined by independent-sample two-tailed *t*-test (*p* < 0.001).

The TOC and n-alkane C_31_ content exhibited similar variations as the sedimentation rate and a significant positive linear correlation was observed (Supplementary Figure 3). The TOC content varied between 0.73 and 1.08 wt% in MIS 3, increased to its maximum value of 1.41 wt% at 1.75 m in MIS 2, and decreased to its minimum value of 0.57 wt% during MIS 1. The n-alkane C_31_ content, a terrestrial biomarker, varied from 96.7–188.1 ng/g in MIS 3, increased to a maximal value of 285 ng/g in MIS 2, and was reduced to its minimum value of 42.1 ng/g during MIS 1. The average TOC and n-alkane C_31_ values during MIS 2 (1.26 ± 0.10 wt% and 224.1 ± 45.44 ng/g, respectively) were significantly higher (*P* < 0.01) than those in MIS 1 (0.68 ± 0.09 wt% and 50.85 ± 8.79 ng/g, respectively) and MIS 3 (0.89 ± 0.11 wt% and 132 ± 33.45 ng/g, respectively). Mean TOC and n-alkane C_31_ values during MIS 3 were significantly higher than those in MIS 1 (*p* < 0.01). The contrast between MIS 1 and MIS 2 was sharper than the transition between MIS 2 and MIS 3, which correlates with more drastic changes in sea level between the MIS 1 and MIS 2 periods. Overall, the sedimentation rate, TOC and n-alkane C_31_ were highest in MIS 2, reflecting maximal terrestrial input.

### Archaeal Community Structure

16S rRNA Illumina tags were clustered into 793 unique OTUs, with an average of 175 OTUs per sample. Good’s coverage values exceeded 99.8%, which is indicative of a high level of diversity coverage in the samples. Alpha diversity indices were calculated using Chao 1 and Shannon (Supplementary Figure 4). Whereas no obvious trend for the Shannon index was observed between the three MISs, there was gradual increase in Chao 1 richness from MIS 3 to MIS 2 and a gradual decrease from MIS 2 to MIS 1.

**FIGURE 3:**
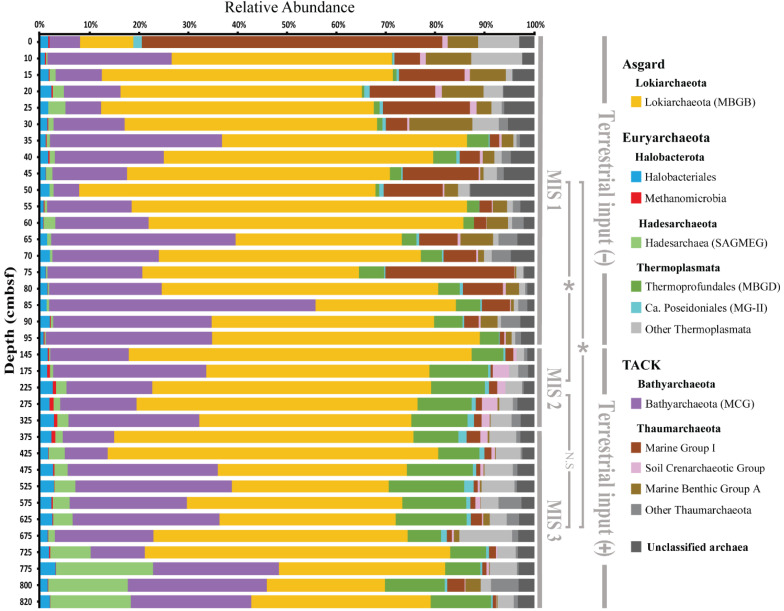
Taxonomic composition of archaeal communities based on 16S rRNA gene sequencing. Samples were divided into MIS 1, MIS 2, and MIS 3, and two stages based on the terrestrial input intensity derived from n-alkane C_31_ [minus sign (–), low terrestrial input; plus sign (+), high terrestrial input]. The similarities were analyzed by ANOSIM (*P < 0.01, N.S = not significant).

*Lokiarchaeota* and *Bathyarchaeota* were consistently the predominant archaeal groups in the core (Figure 3). *Lokiarchaeota* accounted for more than 50% of the archaeal community and *Bathyarchaeota* accounted for approximately 14%. *Thaumarchaeota* were also significant components in the samples. MG-I (*Nitrosopumilales*), the major *Thaumarchaeota* lineage in oceans, accounted for over 60% of archaea in the surface sediments (i.e., 0–5 cm) and the mean was 11.8 ± 14.5% in MIS 1, but only 1.67 ± 0.56% in MIS 2 and MIS 3. MBGA was most prevalent in MIS 1, accounting for 4.34% of the archaeal population on average. A marked decrease in MG-I was observed in MIS 2 and MIS 3; in contrast, the relative abundance of *Thermoprofundales* and *Hadesarchaeota* was higher in sediments deposited during these two stages. The latter two groups on average comprised 10.53 ± 2.87 and 5.71 ± 6.54% of total archaea in MIS 2 and MIS 3, respectively, which were much higher than their presence in MIS 1 (2.70 ± 1.89% for *Thermoprofundales* and 1.02 ± 0.94% for *Hadesarchaeota*). The minor *Thaumarchaeota* group SCG (*Nitrososphaerales*) varied from 0.05 to 3.45% and reached its maximum abundance at 175 cmbsf.

**FIGURE 4:**
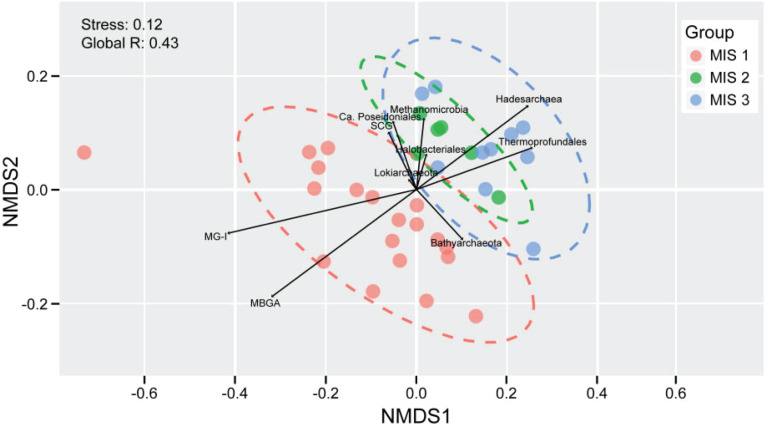
The archaeal community structure in relationship to MIS stages by the NMDS analysis based on Bray-Curtis dissimilarities. Each dot represents a sample and the colors indicated different groups. Ellipses denote 95% confidence intervals.

Based on non-metric multidimensional scaling (NMDS) analysis (Figure 4), MIS 1-derived samples clustered separately from samples from the other periods. This was also supported by pairwise ANOSIM tests (Figure 3).

Redundancy analysis (RDA) was used to identify environmental factors that correlate with the archaeal community structure. As displayed in Supplementary Figure 5, the first and second canonical axes represented 79.9% (26.2% of total) and 19.5% (6.4% of total) of the total variance observed, respectively. RDA1 separated MIS 1 archaeal communities from MIS 2 and MIS 3 communities. The n-alkane C_31_ (*p* < 0.01) and TOC (*p* < 0.01) correlated significantly with the archaeal communities and were highest in MIS 2 and MIS 3. While TOC was significantly correlated with *Thermoprofundales* and total nitrogen correlated best with SCG, n-alkane C_31_ was closely related to *Methanomicrobia*, *Ca*. Poseidoniales, and *Hadesarchaeota*. Additionally, MG-I and MGBA negatively correlated with TOC and n-alkane C_31_. Notably, *Lokiarchaeota* and *Bathyarchaeota*, the two most abundant groups in the sediment core, did not correlate with TOC or n-alkane C_31_.

### The qPCR Abundance of 16S rRNA Genes of Archaea and AOA

The archaeal 16S rRNA gene copy number per gram of wet sediment (/g.w.sdmt) ranged from 1.89 × 10^5^ to 2.58 × 10^6^ in MIS 3, and it gradually increased in MIS 2 followed by a decrease in MIS 1 (Supplementary Figure 6A). The highest gene copy number (8.39 × 10^6^/g.w.sdmt) was found at 175 cmbsf, consistent with the highest concentration of TOC. Overall, the mean archaeal 16S rRNA gene copy numbers were 8.32 × 10^5^/g.w.sdmt in MIS 1, 4.27 × 10^6^/g.w.sdmt in MIS 2 and 1.02 × 10^6^/g.w.sdmt in MIS 3. The SCG 16S rRNA gene copy number showed a similar trend with that of archaea but was orders of magnitude lower. The SCG 16S rRNA gene copy number varied between 6.46 × 10^2^/g.w.sdmt and 1.70 × 10^4^/g.w.sdmt (Supplementary Figure 6B). While a large variation occurred between 50 and 100 cm during MIS 1, it reached the maximum value of 3.31 × 10^5^/g.w.sdmt at 175 cmbf in MIS 2 and then decreased gradually to the lowest value of 1.82 × 10^3^/g.w.sdmt in MIS 3. On average, the SCG gene copy number was 3.63 × 10^3^/g.w.sdmt in MIS 1, 7.59 × 10^4^/g.w.sdmt in MIS 2 and 7.41 × 10^3^/g.w.sdmt in MIS 3.

MG-I gene copy number exhibited a different pattern. MG-I gene copy number showed a decreasing trend in MIS 1, with three peaks at 35, 65 and 85 cmbsf. It decreased downward into MIS 2 and MIS 3 with the lowest value of 2.14 × 10^3^/g.w.sdmt at 475 cmbsf in MIS 3. On average, the MG-I gene copy number was 5.50 × 10^4^/g.w.sdmt in MIS 1, 4.79 × 10^4^/g.w.sdmt in MIS 2 and 1.48 × 10^4^/g.w.sdmt in MIS 3.

### Impact of Terrestrial Input on SCG and Thermoprofundales

Five SCG OTUs with the highest relative abundances (OTU321, OTU463, OTU650, OTU530, and OTU593) showed a drastic increase during MIS 2 (Supplementary Figure 7A), suggesting that they may be derived from terrestrial sources. These OTUs displayed a consistent pattern of fluctuation, suggesting that terrestrial input may be a significant factor shaping their distribution. The distinct patterns of SCG OTUs present in MIS 1 and MIS 3 may indicate varying influence of different climatic factors on SCG sedimentation, *in situ* growth, and/or preservation during periods of high and low terrestrial input. Additionally, the absolute abundance of SCG was significantly correlated with n-alkane C_31_ (*R* = 0.61), TOC (*R* = 0.63), and sedimentation rate (*R* = 0.44) in the sediment core, suggesting that it is of terrestrial origin (Supplementary Figure 8A).

SCGs in marine sediments consisted of OTUs −303, −42, −453, −593, −463, −321, −74, −193, −650, −783, −530, −594, −230, −494, −274, and −45. Interestingly, phylogenetic analysis demonstrated that SCG sequences obtained from marine and terrestrial environments were not phylogenetically distinct (Supplementary Figure 9).

The absolute abundance of *Thermoprofundales* also showed significant positive correlations with n-alkane C_31_ (*R* = 0.54), TOC (*R* = 0.58), and sedimentation rate (*R* = 0.51) (Supplementary Figure 8B). Further investigation showed that the seven most abundant *Thermoprofundales* OTUs exhibited a marked increase in MIS 2-derived samples (Supplementary Figure 7B). The results indicated that *Thermoprofundales* was significantly affected by terrestrial input. Overall, the distributional patterns of *Thermoprofundales* OTUs were similar to SCG OTUs, but with orders of magnitude higher abundance.

Interestingly, the ratio of sulfate to chloride was consistently around 0.05 in the MIS 1-derived sediments (Supplementary Figure 2), suggesting that there was no significant loss of sulfate through dissimilatory sulfate reduction. The ratio decreased gradually with increasing depth in MIS 2- and MIS 3-derived sediments, suggesting sulfate reduction in the deeper sediments. However, no known sulfate-reducing archaea (SRA) were found. The profiles could be caused by sulfate-reducing bacteria (SRB) or unknown SRA. We examined the bacterial data and indeed found presence of SRB in all the sediment layers (present in very low abundance). However, unlike archaea, the distributional patterns of bacteria, including SRB, were unrelated to the terrestrial input (Supplementary Figure 10).

### Network Analysis of Archaeal Communities

**FIGURE 5:**
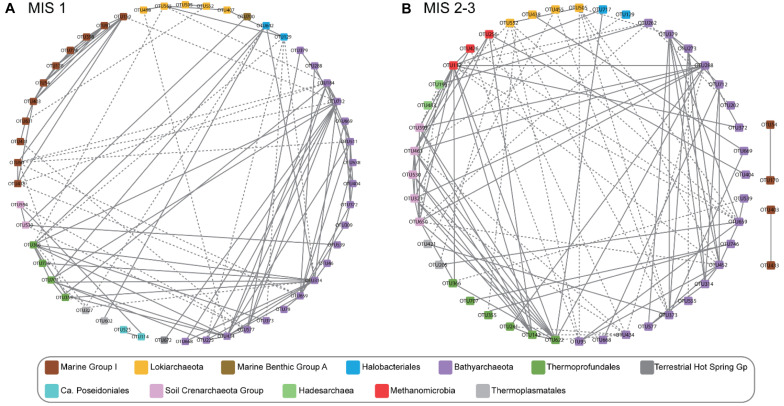
Correlation network analysis of archaeal communities. Each node represents an OTU and edges are defined based on the correlation between abundance profiles across samples in MIS 1 **(A)** and MIS 2-MIS 3 **(B)**. Nodes with significant correlations are connected, with solid and dotted lines representing positive and negative correlations, respectively. Nodes are colored based on taxonomy.

Both ANOSIM analysis and NMDS ordination analysis clearly separated samples into two groups: MIS 1 and MIS 2 – MIS 3 (Figures 3, 4). As such, we constructed archaeal co-occurrence networks to investigate the correlations between the OTUs within MIS 1 (Figure 5A), and OTUs shared between MIS 2 to MIS 3 (Figure 5B). A highly connected cluster (module) of MG-I OTUs was observed in the MIS 1 group, whereas only four MG-I OTUs with two degrees were observed in the MIS 2 – MIS 3 group. In contrast, SCG OTUs exhibited an opposite pattern. SCG OTUs were less connected in the MIS 1 group but intensively connected within the group and with benthic groups such as *Bathyarchaeota* and *Thermoprofundales* in the MIS 2 – MIS 3 group. Additionally, *Methanomicrobia* were more intensely connected in the MIS 2 – MIS 3 group compared to the MIS 1 group. *Lokiarchaeaota*, one of the “Asgard archaea,” showed less connection to other archaeal groups although they were present in high abundance, which is apparently contradictory to the syntrophic nature of the only lab-cultivated member of the group, ‘*Candidatus* Prometheoarchaeum syntrophicum’ (Imachi et al., 2020). However, since co-correlations were not computed between archaea and bacteria, it is possible that the *Lokiarchaeota* could be syntrophic with one or more bacteria.

## Discussion

### Geological Framework of the Pearl River Submarine Canyon Sediments

The sediment core from the Pearl River Submarine Canyon region of the South China Sea (Figure 1) represented three MISs, which were correlated with changes in sedimentation rate, TOC and the terrestrial n-alkane C_31_ (Figure 2). MIS 2, the glacial period, displayed the highest levels of TOC, n-alkane C_31_ and sedimentation rate while MIS 1 (Holocene) displayed the lowest of the three parameters. High enrichment of organic carbon could be caused by either an increased input of organic matter or increased preservation in anoxic marine sediments (Stein, 1990). The significant positive correlation between organic carbon and sedimentation rate (Supplementary Figure 3) suggests that the former is the more important, although fine-grained and reducing environments could contribute to the preservation of organic matter (Müller and Suess, 1979). Several studies have shown that OM can be stabilized and can sustain long-term preservation in marine sediments by binding with metal ions and clay minerals (Curry et al., 2007; Blattmann et al., 2019).

Notably, the greatest decrease in relative sea level was observed in the transition from MIS 2 to MIS 1 compared to MIS 3 to MIS 2. The observations made in MIS 2 support previous evidence that global cooling is associated with accumulation of organic carbon in the deep sea, and OM fluxes to the deep sea have been estimated to be ∼50% higher during glacial maxima than during interglacials (Cartapanis et al., 2016). Moreover, there have been prior reports of a link between global cooling and accelerated terrestrial sedimentation during the late Cenozoic Era (Molnar, 2004), though this relationship has been challenged (Willenbring and von Blanckenburg, 2010). Additionally, the paleo Pearl River Estuary extended outward and contracted inward with the large eustatic sea-level fall and rise (Figure 1). Although different evolutionary models of the Pearl River Estuary have been proposed (Wu et al., 2007; Tang et al., 2010), they agree that geographical changes in the estuarine system altered the terrestrial input, sedimentary processes or depositional environments. Understanding of the geological framework is important for interpreting changes in the archaeal community structure in the Pear River Submarine Canyon sediments (see below).

### The Impact of Terrestrial Input on Archaeal Communities

By profiling the vertical distribution of archaeal communities and cross-referencing environmental footprints conserved in the sediment, our understanding of the factors that shape archaeal structure is improved. For example, while pH and temperature have been deemed important regulators of community structure in non-saline environments (Cole et al., 2013; Hedlund et al., 2016; Wen et al., 2017), organic carbon concentration has been shown to be an important factor shaping archaeal community structure in marine sediments (Durbin and Teske, 2012; Learman et al., 2016). In other deep-sea ecosystems, bottom water temperatures and trophic resources including organic substrates (Danovaro et al., 2016) at least partially drive habitat preferences. Our results show that the alteration of archaeal community structure in marine sediments is associated with terrestrial input, either directly, by bringing in compounds such as long-chain n-alkanes derived from epicuticular waxes of vascular plant leaves (Bi et al., 2005), which may stimulate or support the growth of some lineages, or indirectly, by changing the depositional conditions.

In particular, the occurrence of the highest total abundance of the archaeal 16S rRNA gene in MIS 2-derived samples (Supplementary Figure 6A) can be explained in detail. One explanation is that fermentation of a large amount of terrestrial organic matter occurred *in situ*, which provided energy for the growth of archaea that may have participated in the fermentation process. Recent research showed that fermentation-related genes encoding multiple fermentation pathways were found in marine sediments (Zinke et al., 2019). Additionally, fermentation pathways have been found in genomes of *Thermoprofundales* (Zhou et al., 2019) and *Lokiarchaeota* (Orsi et al., 2020). The recently cultivated member of the *Lokiarchaeota*, ‘*Candidatus* Prometheoarchaeum syntrophicum’, has been shown to ferment amino acids (Imachi et al., 2020). Some archaeal lineages are capable of degrading refractory organics (He et al., 2016; Yu et al., 2018), which are further utilized by other organisms.

Another explanation is that the increased supply of terrigenous matter may also bring in nutrients that stimulated phytoplankton growth, which produced rich sources of labile organic carbon such as amino acids. That labile carbon pool may have settled rapidly to the sea floor due to the high sedimentation rate and became trapped in fine-grained sediments (Curry et al., 2007), with labile fractions used by heterotrophic microbes, including archaea.

The low-abundance archaeal group SCG, important AOA that dominate soil archaeal communities (Bates et al., 2011), was strongly associated with long-chain n-alkane C_31_ (Supplementary Figure 8A), supporting its terrestrial origin (Seki et al., 2003). In accordance with this hypothesis, SCG OTUs from this study exhibited a high similarity with SCG from terrestrial environments based on phylogenetic analysis (Supplementary Figure 9). While fluctuations were apparent across all SCG OTUs throughout the sediment core, all five SCG OTUs (321, 463, 650, 530, and 593) simultaneously increased dramatically during the Last Glacial Maximum, when terrestrial input was at its maximum (Supplementary Figure 7A), which would also transport a larger amount of TerrOC into the ocean than during interglacial periods.

The presence of terrestrial SCG in marine sediment may also suggest that they survive and remain active after being transported from soil. Several studies have shown that SCG have diverse metabolisms, including autotrophy (Stieglmeier et al., 2014) and heterotrophy (Hallam et al., 2006; Mußmann et al., 2011). An *in situ* cultivation experiment in deep-sea sediments indicated SCG was among the most abundant group in the initial archaeal community and they still existed after 405 days (Takano et al., 2010). RDA analysis showed that SCG was the lineage most closely related to total nitrogen (Supplementary Figure 5), which might indicate its role in metabolizing organic nitrogen (Di et al., 2010; Levičnik-Höfferle et al., 2012; Lehtovirta-Morley et al., 2016; Abby et al., 2018). Network analysis showed not only intimate interactions within SCG OTUs but also with other benthic groups such as *Bathyarchaeota*, *Thermoprofundales*, and *Methanomicrobia* (Figure 5B). Such interactions could result from metabolic associations. For example, the production of urea by *Bathyarchaeota* (Pan et al., 2020) could be used by SCG (Tourna et al., 2011) and the synthesis of cobalamin by *Nitrososphaera* (Lu et al., 2020), a group of SCG, could stimulate growth of cobalamin-dependent lineages. However, we cannot exclude the possibility that interactions could be caused by their similar environmental niches. Interestingly, SCG have been reported to grow in the absence of nitrification (Jia and Conrad, 2009).

Another intriguing possibility for the existence of SCG in sediment samples and enrichment in MIS 2-derived sediments is their preference for high ammonia concentrations. It has been reported that SCG prefers ammonia-rich environments (Prokopenko et al., 2006; Tourna et al., 2011). MIS 2-derived sediments received a significant increase in organic matter (Figure 2), and a large amount of ammonium could be produced due to the ammonification and mineralization of the organic matter (Prokopenko et al., 2006). Further research should be undertaken to investigate the activity of the SCG lineage in marine sediments considering its potential roles in the nitrogen and carbon cycles.

16S rRNA gene copy numbers of *Thermoprofundales*, showed significant positive correlation with TOC, n-alkane C_31_, and sedimentation rate, similar to SCG (Supplementary Figure 8B). Although *Thermoprofundales* were initially found in marine sediments, they are spread across a wide range of terrestrial habitats including soil, wetland soil, and inland lakes (Jiang et al., 2008; Zhou et al., 2019). The observed increase of *Thermoprofundales* in MIS 2 may partly be explained by the input of terrestrial *Thermoprofundales* lineages along with enhanced terrestrial input. In line with this, numerous subgroups of *Thermoprofundales* were found in both saline and non-saline environments (Zhou et al., 2019). Seven *Thermoprofundales* OTUs (OTU142, OTU355, OTU384, OTU707, OTU622, OTU366, and OTU776) closely tracked terrestrial input (Supplementary Figure 7B). Another possibility is that *Thermoprofundales* abundance may be indirectly impacted by terrestrial TOC. Indeed, this is in keeping with research that indicates the critical role of *Thermoprofundales* in the sedimentary carbon cycle (Lloyd et al., 2013; Vigneron et al., 2014; Zhou et al., 2019).

Although *Lokiarchaeota* were identified as the dominant archaea with 50% representation, redundancy analysis revealed that these organisms were not correlated with organic carbon or n-alkane C_31_ (Supplementary Figure 5). The cultivated member of the *Lokiarchaeota* is extremely slow-growing in lab cultures (Imachi et al., 2020) and consequently they may be less sensitive to organic carbon. Similarly, *Bathyarchaeota*, though counting for a large proportion (14%) of the total archaeal population, did not show significantly correlations with organic carbon or n-alkane C_31_, also likely due to their slow growth (Yu et al., 2018).

**FIGURE 6:**
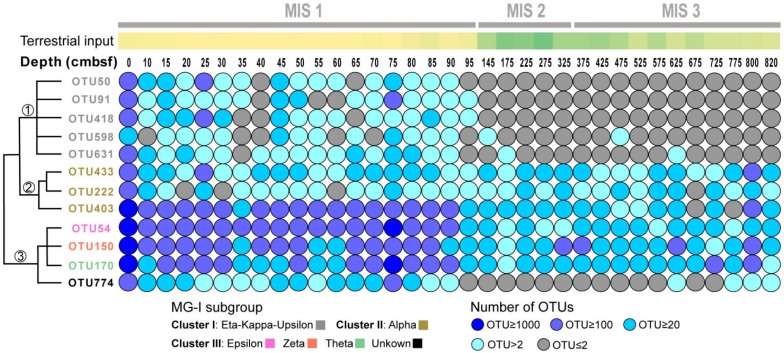
The depth profiles of 12 MG-I OTUs having the highest mean abundance. The color bar above represents the intensity of terrestrial input (yellow: low; green: high). Colored squares on the bottom left represent different MG-I subgroups. Circles in different colors on the bottom right represent the number of OTUs identified. The cluster tree on the left was simplified schematic of evolutionary relationship according to the phylogenetic tree (Supplementary Figure 9).

The sources of MG-I in Pearl River Submarine Canyon sediments may be complicated. MG-I recovered mainly from marine sediments include ε (epsilon), ζ (zeta), θ (theta), η (eta), κ (kappa), υ (upsilon) and ι (iota) subgroups, whereas γ (gamma), δ (delta) and β (beta) subgroups are usually identified in water columns and the α (alpha) subgroup is found in both water column and sediment (Durbin and Teske, 2010; Lauer et al., 2016). For example, MG-I subgroups ε, κ, and υ are predominant in the subseafloor sediments at ODP Site 1225 (Lauer et al., 2016). MG-I populations shifted from multiple subgroups to the dominance of subgroups υ and η as the sediment depth increases in the South Pacific Gyre (Durbin and Teske, 2010). Our results showed two distributional patterns of MG-I subgroups: the ε-ζ-θ subgroups were present across all depth intervals and the η-κ-υ subgroups mainly existed in the MIS 1 stage. The α subgroup may be a mixture of planktonic or benthic MG-I populations (Figure 6), whose presence is likely linked to the availability of oxygen or nitrate in the sediment porewater (Jorgensen et al., 2012; Lauer et al., 2016; Hiraoka et al., 2020).

MBG-A, a sister group of MG-I, mainly existed in the MIS 1-derived sediments and negatively correlated with the terrestrial biomarker n-alkane C_31_ (Supplementary Figure 5). *Methanomicrobia*, dominant in methane-containing sediments (Hamdan et al., 2013), showed a positive correlation with terrestrial input. Depth-related increases in the abundance of *Hadesarchaeota* have been previously characterized in the South China Sea (Cui et al., 2016) and it exhibited a weak positive correlation with terrestrial input. Ca. *Poseidoniales* is the dominant planktonic archaeal group in ocean surface waters (Zhang et al., 2015; Rinke et al., 2019) and the lineage showed a weak correlation with terrestrial signal, suggesting pelagic archaea could be trapped with advanced sedimentation.

## Conclusion

This study offers a unique look at archaeal communities in a deep-sea sediment column spanning over 59 k years in the Pearl River Submarine Canyon. While it is well-known that archaea in deep sea ecosystems play critical roles in biogeochemical cycling, much remains unknown about their specific metabolic capacities and environmental adaptation in deep sea sediments. In addition to broad characterization of a sediment core, this paper provided an in-depth analysis of diverse archaeal groups in marine sediments in the context of terrestrial organic input in the South China Sea. The SCG archaea, though a minor group, revealed high correlation with terrestrial input, which may be a consequence of its terrestrial origins coupled with high sedimentation of organic carbon. MG-I, many of which can be indigenous AOA in marine environments, may participate in nitrogen cycling in the upper layer of sediments. Terrestrial input correlated negatively with MG-I and may insert adaptive pressures to the group. *Thermoprofundales* may be directly impacted by terrestrial input or indirectly influenced via organic carbon. Additionally, *Hadesarchaeota*, and MBGA could also be affected by terrestrial input directly or directly.

These results demonstrate that composition of benthic archaea in the South China Sea may be controlled by glacial-interglacial cycles that control terrestrial organic input, providing a plausible link between global climate cycles and microbial population dynamics in deep-sea marine sediments. Though genomic analysis provided important findings, the broad metabolic capacities of some of these archaeal groups are lacking and their roles in deep sea sediments should be pursued.

## Data Availability Statement

The datasets presented in this study can be found in online repositories. The names of the repository/repositories and accession number(s) can be found in the article/Supplementary Material.

## Author Contributions

DL, PW, WX, and CZ contributed to experimental design. DL, WX, and JL completed laboratory work. DL contributed to bioinformatics work. DL wrote the manuscript. BH, PW, WX, TP, and CZ contributed to the writing, revision, and final polishing of manuscript. All authors contributed to the article and approved the submitted version.

## Conflict of Interest

The authors declare that the research was conducted in the absence of any commercial or financial relationships that could be construed as a potential conflict of interest.

## Footnotes

1 http://drive5.com/uparse/

2 http://rdp.cme.msu.edu/
