# Supplementary material for: Impact of Terrestrial Input on Deep-Sea Benthic Archaeal Community Structure in South China Sea Sediments
Source: Front Microbiol. 2020 Nov 5;11:572017. doi: 10.3389/fmicb.2020.572017 (PMC7674655; doi:10.3389/fmicb.2020.572017)
Supplement: Supplementary file 1 [file Data_Sheet_1.docx]

Supplementary Material

**Figure S1.** Rarefaction curves of the Shannon diversity of archaea (A) and the overserved OTUs of archaea (B).

**Figure S2.** Depth profiles of sulfate (blue diamond), chloride (blue triangle), and sulfate to chloride ratio (yellow circle).

**Figure S3.** The correlation of TOC (blue) or C31 (green) with sedimentation rate, respectively.

**Figure S4.** The Chao 1 richness and Shannon index of archaea with depth. The shaded area represents minimum-maximum range.

**Figure S5.** Distance-based redundancy analysis (db-RDA) of the relationship between environmental factors (red lines) and archaeal communities (blue lines). The asterisk stands for significant correlation (p<0.01). WC: water content; C31: C31 n-alkane; ^13^C: δ^13^C (OC); TN: total nitrogen; TOC: total organic carbon. The color code is consistent with that in Figure 4.

**Figure S6.** The quantification of archaeal 16S rRNA gene copies (A) and AOA groups (B) from different depth intervals. Error bars indicate 95% confidence intervals. The asterisks indicate only two biological replicates for a sample. The abundance was calculated by multiplying their respective relative abundance by the total number of 16S rRNA gene copies.

**Figure S7.** The depth profiles of absolute abundance of SCG OTUs (A) and *Thermoprofundales* OTUs (B).

**Figure S8.** Linear correlation plots showing the relationship between log10-transformed gene copy abundance for SCG (A) and *Thermoprofundales* (B) with C31 n-alkane, TOC, and sedimentation rate, respectively.

**Figure S9.** The phylogenetic tree of MG-I and SCG. For MG-I, the color of inner circle represents the source (dark blue: marine sediments; light blue: marine water). For SCG, the color of inner circle denotes the locations identified (sky blue: marine environments; grey: terrestrial environments).

**Figure S10.** Taxonomic composition of bacterial communities based on 16S rRNA sequencing. Comminity dissimilarities using the Bray-Curtis metric were analyzed by ANOSIM (*P<0.05, N.S = not significant).

**Table S1.** Environmental parameters of MD12-3433.

**Table S2.** Real-time quantitative PCR of the 16S rRNA gene (logarithmic transformation).

**Fig. S1**


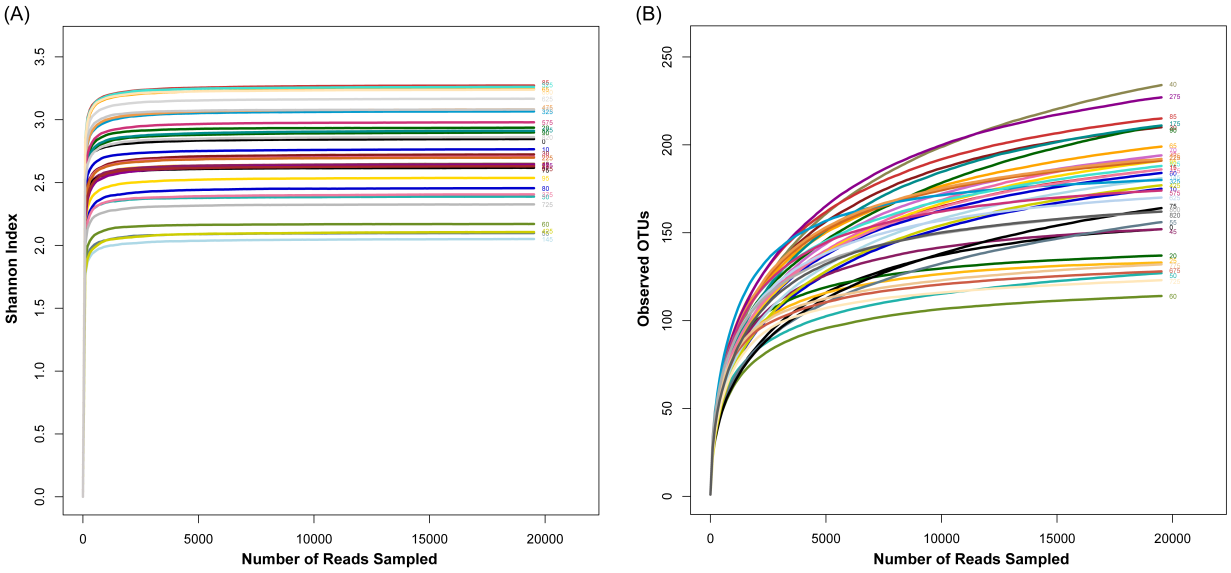


**Fig. S2**


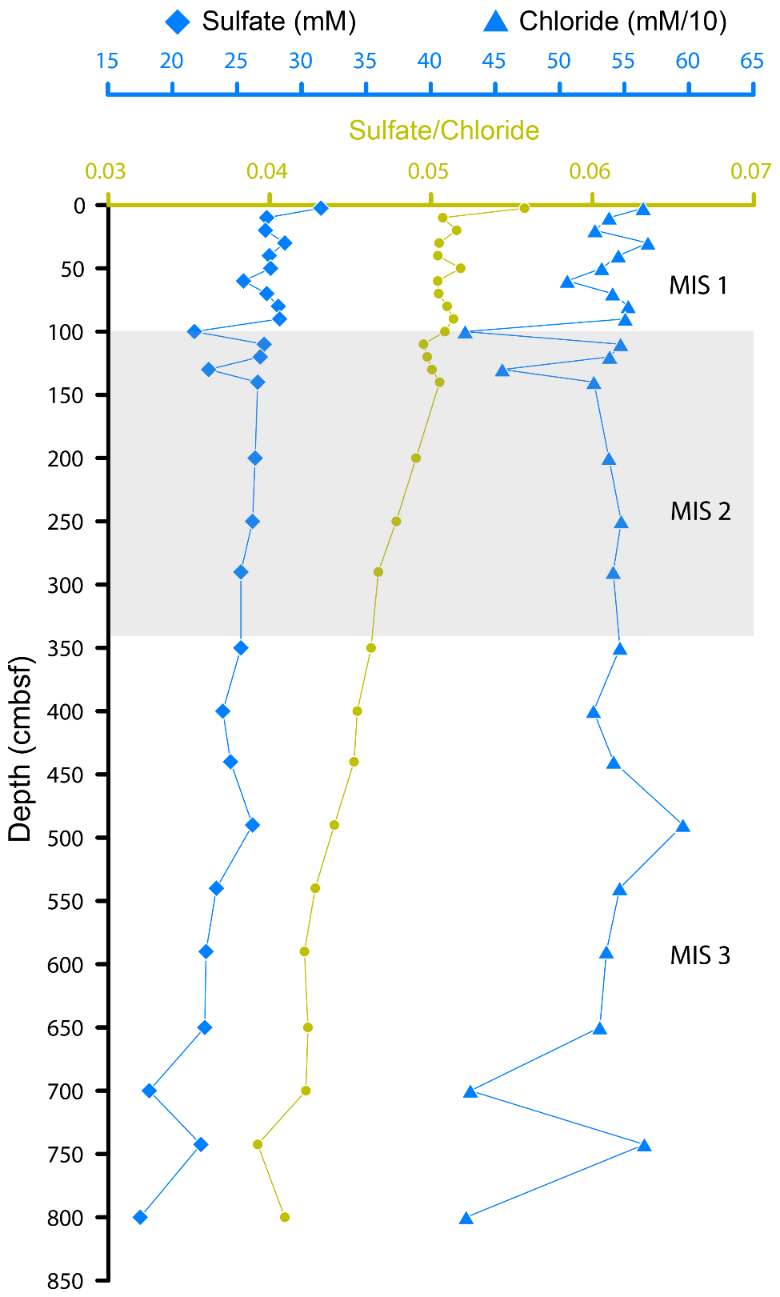


**Fig. S3**


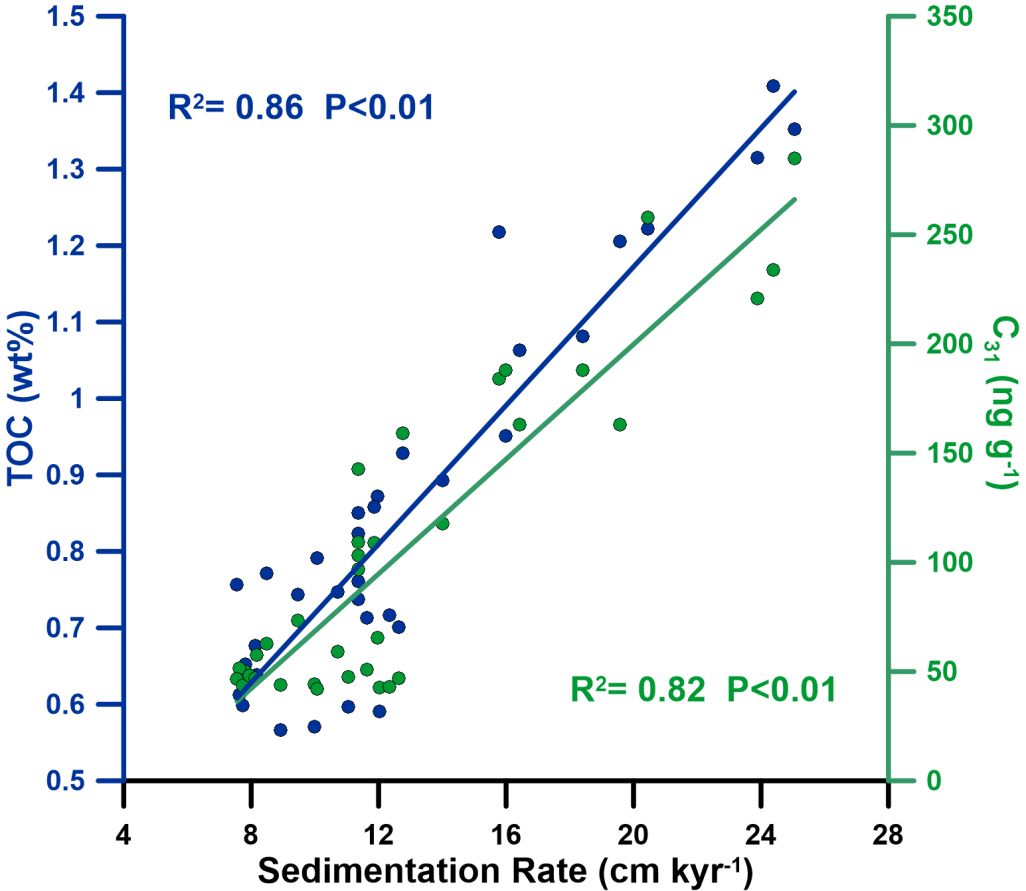


**Fig. S4**


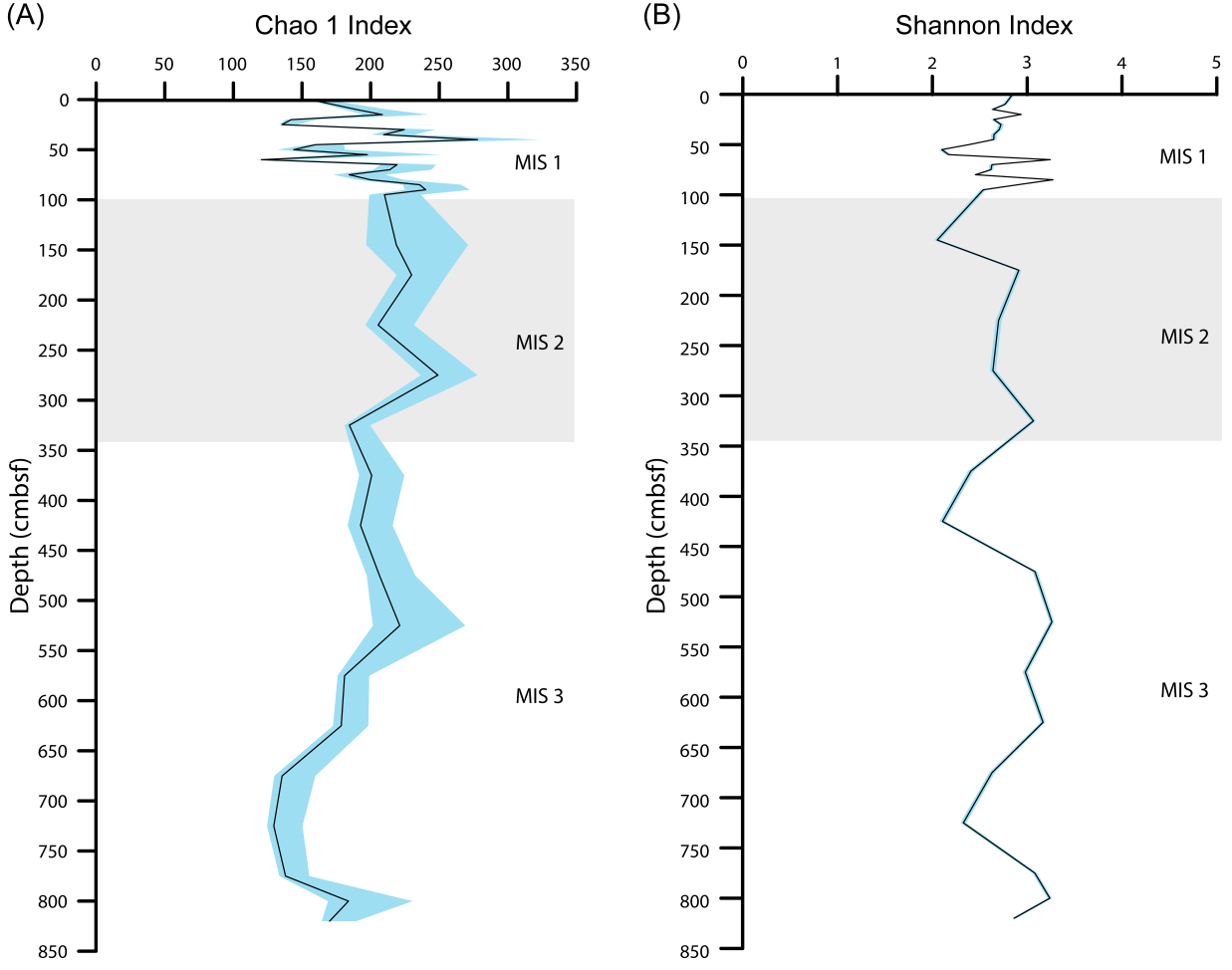


**Fig. S5**


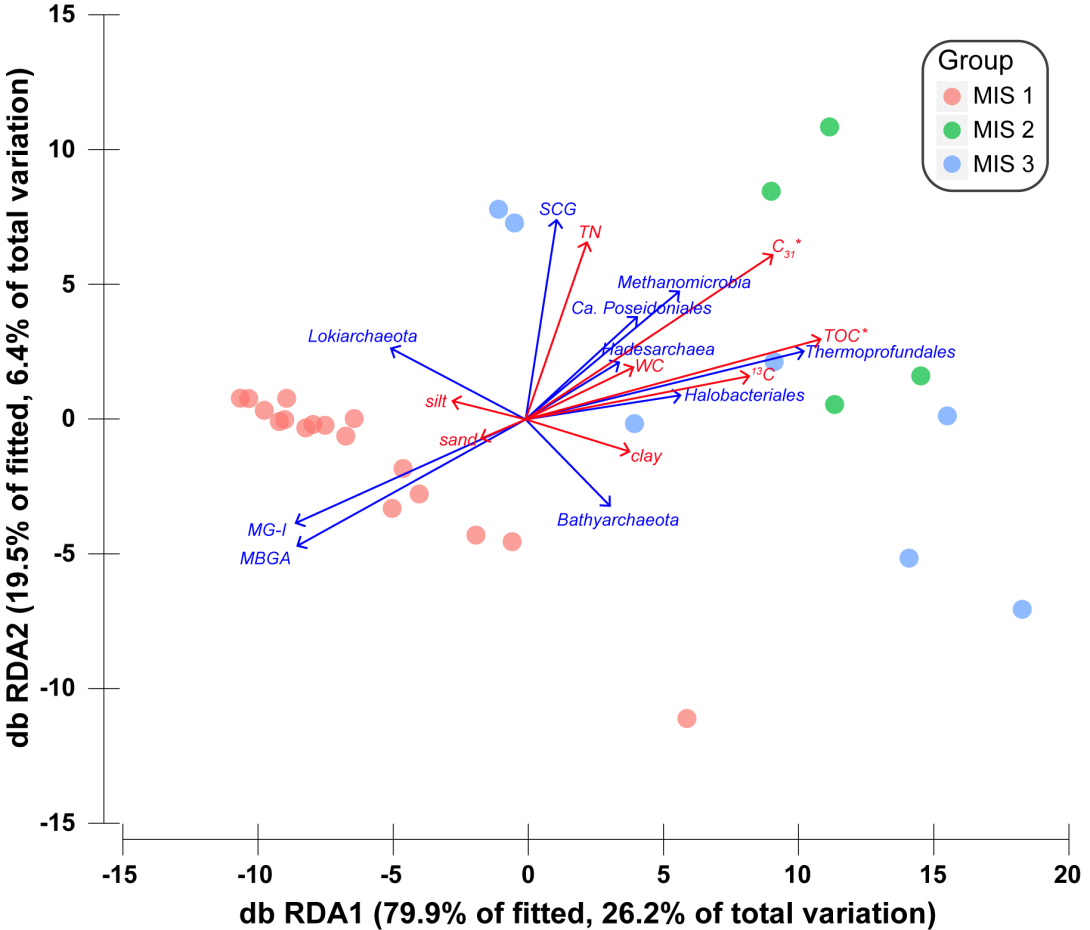


**Fig. S6**


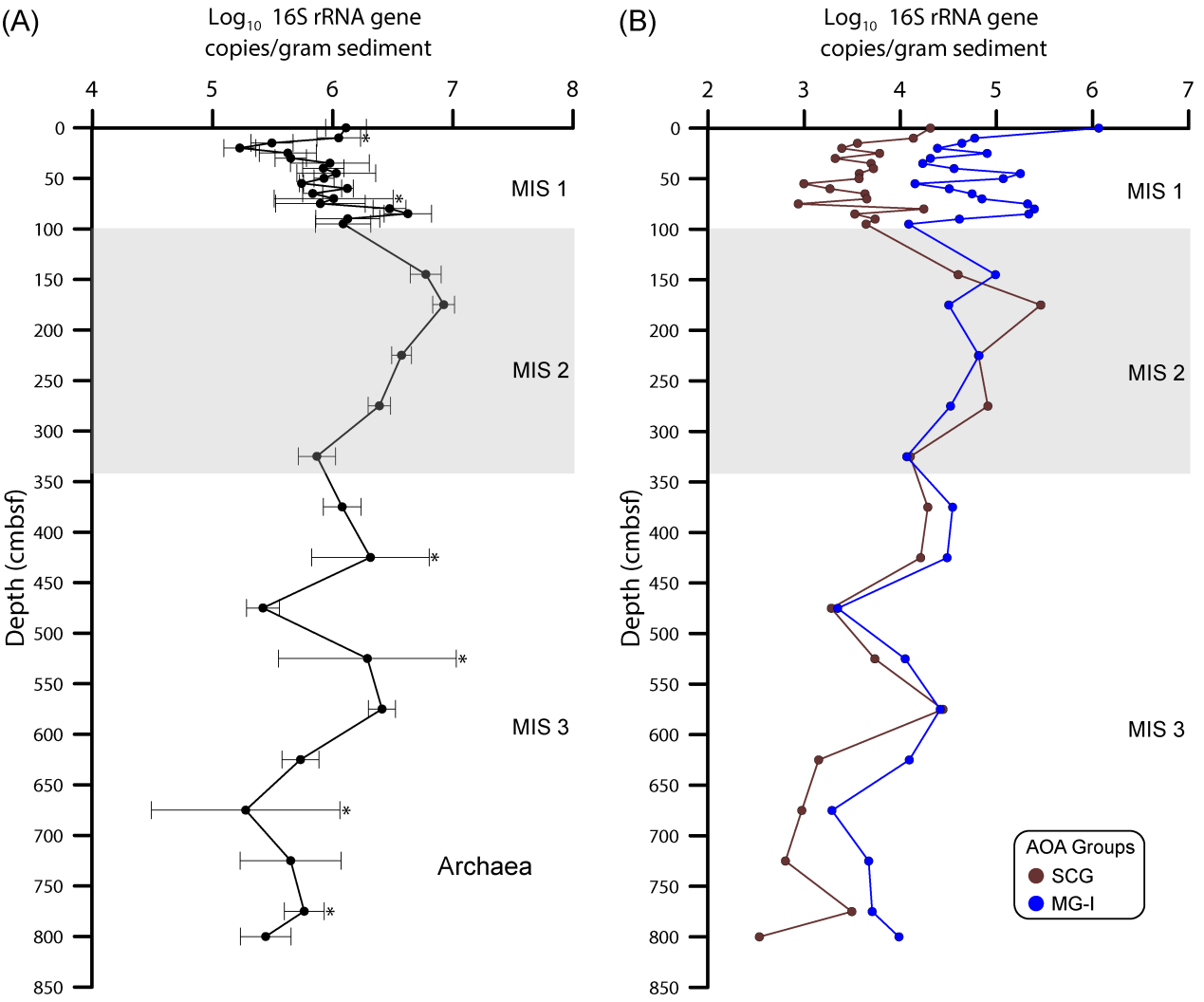


**Fig. S7**


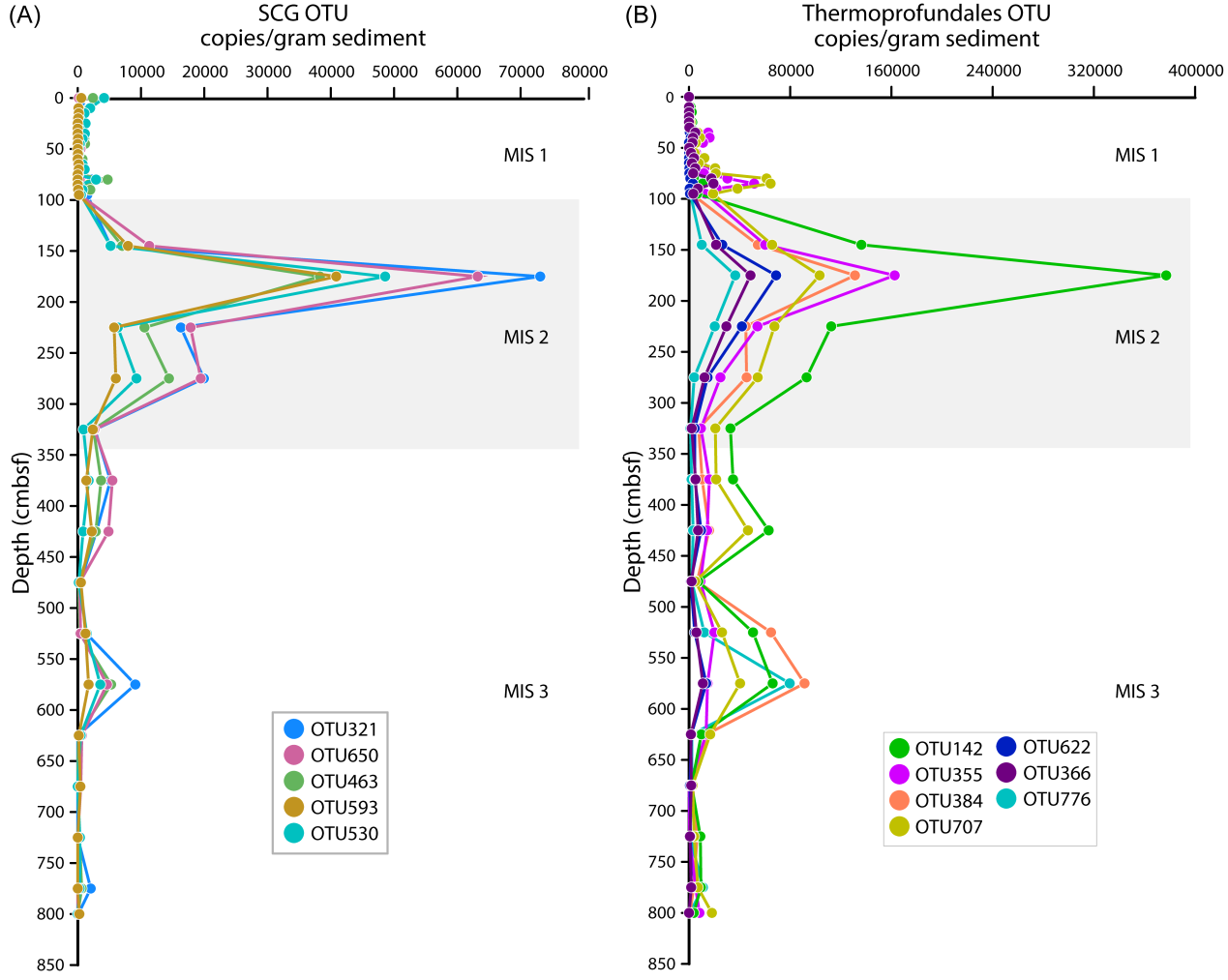


**Fig. S8**


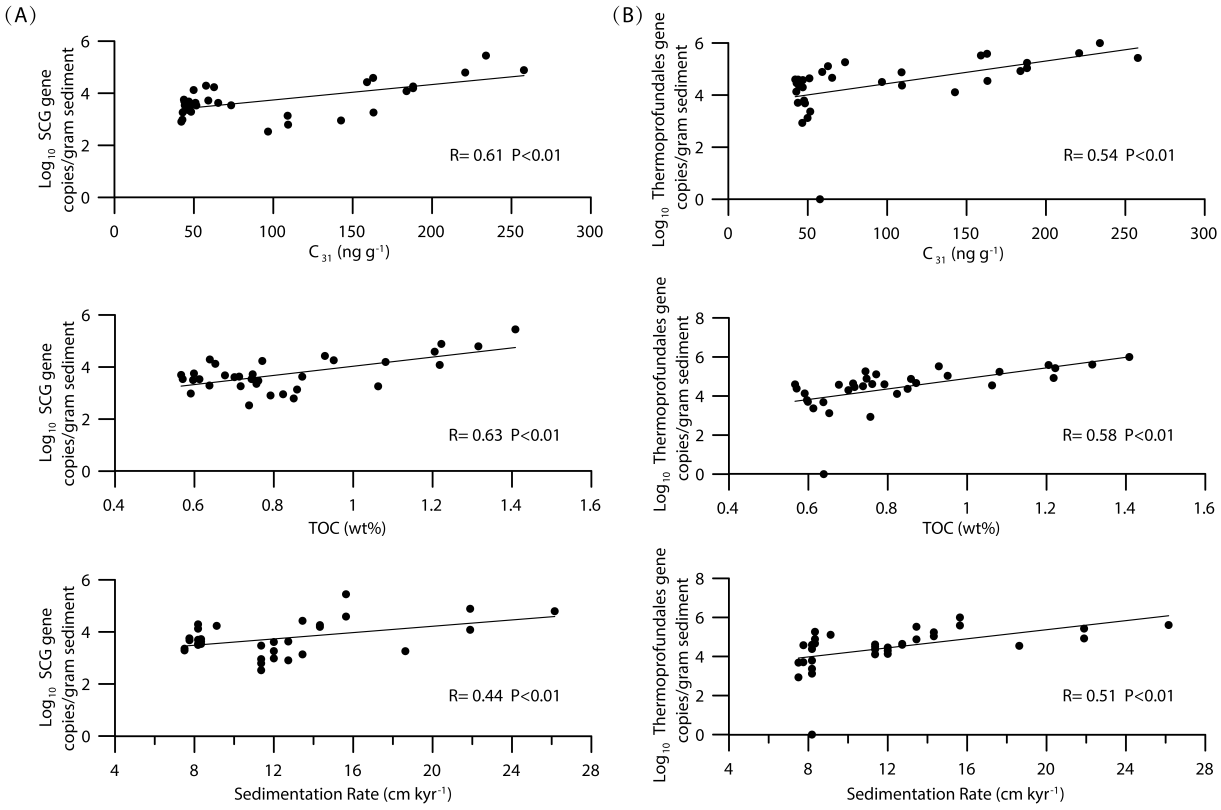


**Fig. S9**


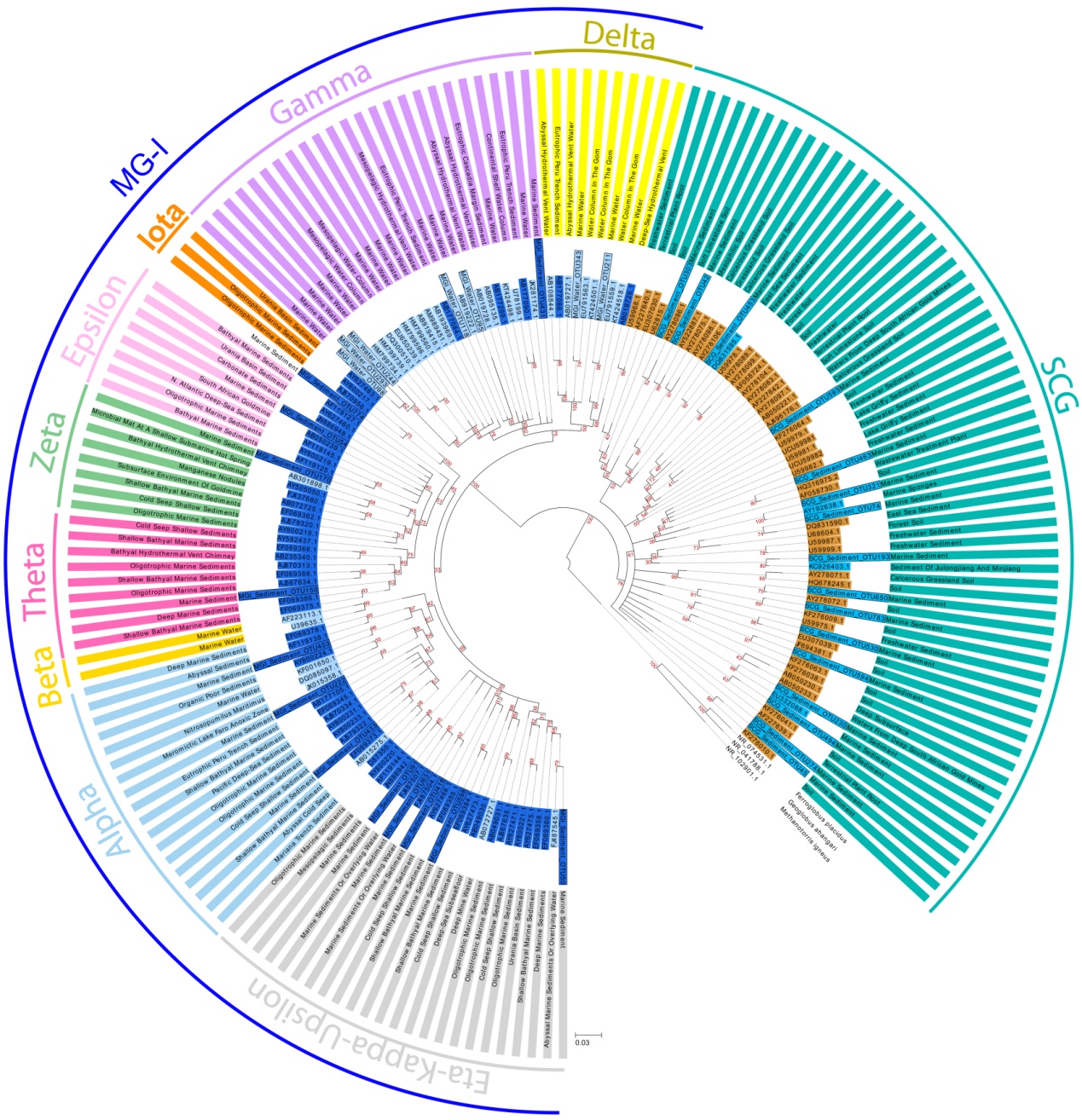


**Fig. S10**

**
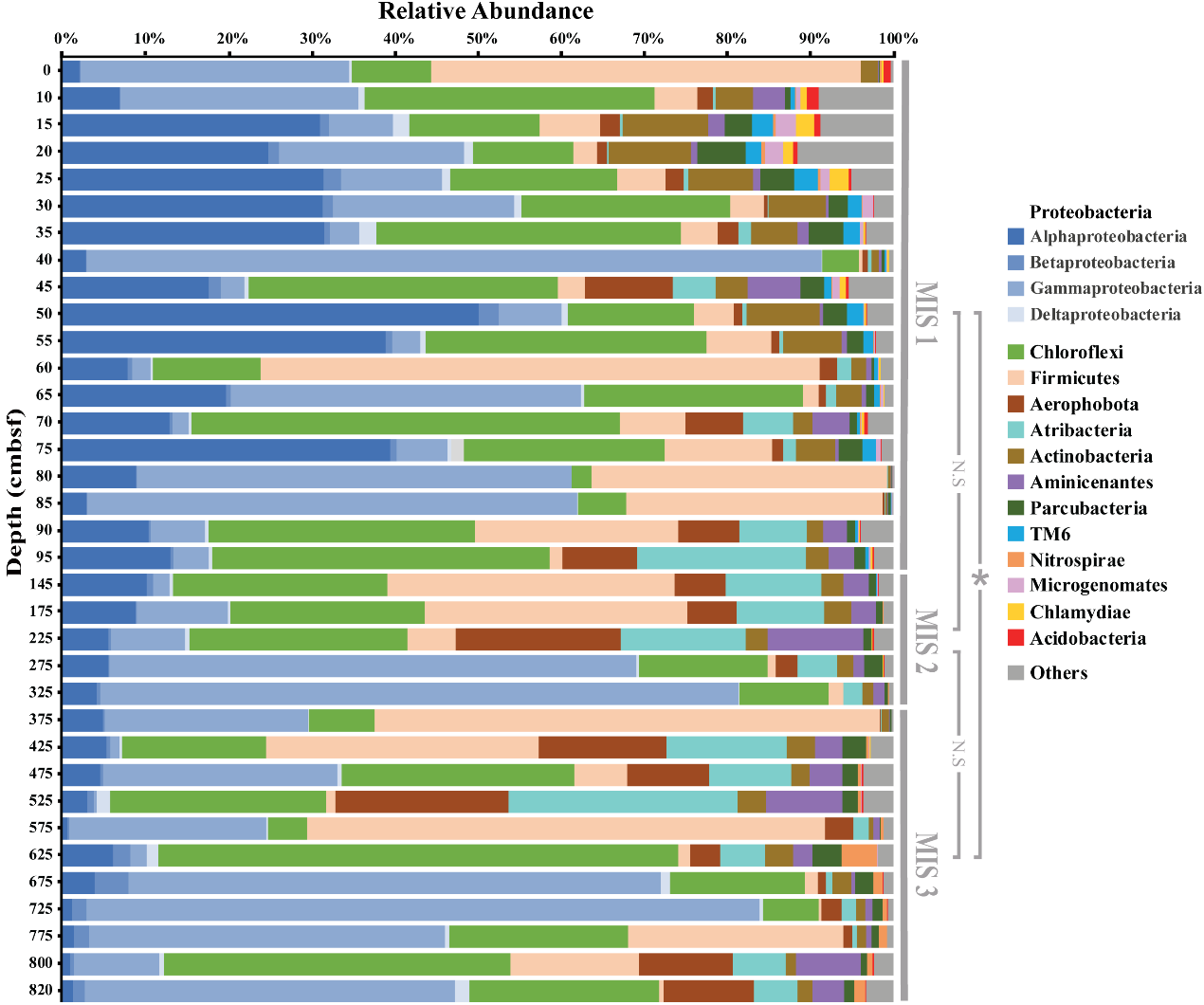
**

**Table S1**

| Depth (cm) | Total Organic Carbon  (wt%) | δ^13^C_OC_  (‰) | clay (%) | silt  (%) | sand  (%) | TN (wt%) | Water Content  (%) | n-alkane C_31_  (ng/g) |
| --- | --- | --- | --- | --- | --- | --- | --- | --- |
| 0 | 0.64 | -21.01 | N/A | N/A | N/A | 0.18 | N/A | 57.64 |
| 10 | 0.65 | -21.15 | 12.77 | 74.94 | 12.28 | 0.16 | 49.27 | 49.83 |
| 15 | 0.61 | -21.13 | 11.56 | 73.18 | 15.26 | 0.16 | 45.92 | 51.53 |
| 20 | 0.76 | -21.15 | 14.91 | 76.89 | 8.21 | 0.19 | 25.24 | 46.52 |
| 25 | 0.60 | -21.18 | 15.06 | 77.53 | 7.38 | 0.16 | 31.27 | 43.71 |
| 30 | N/A | N/A | N/A | N/A | N/A | N/A | N/A | 48.22 |
| 35 | 0.68 | -21.21 | 14.08 | 76.34 | 9.58 | 0.17 | 34.18 | 46.82 |
| 40 | 0.57 | -21.18 | 48.62 | 46.85 | 4.54 | 0.15 | 44.36 | 43.92 |
| 45 | 0.57 | -21.23 | 44.95 | 51.03 | 4.02 | 0.15 | 39.55 | 44.24 |
| 50 | 0.60 | -21.02 | 49.52 | 46.81 | 3.66 | 0.15 | 41.27 | 47.64 |
| 55 | 0.59 | -21.17 | 10.75 | 78.46 | 10.79 | 0.15 | 38.93 | 42.76 |
| 60 | 0.72 | -21.23 | 12.64 | 73.62 | 13.75 | 0.16 | 36.07 | 42.95 |
| 65 | 0.70 | -21.05 | 14.10 | 78.09 | 7.82 | 0.16 | 38.80 | 46.82 |
| 70 | 0.71 | -21.14 | 55.17 | 39.34 | 5.47 | 0.14 | 40.95 | 51.01 |
| 75 | 0.79 | -21.23 | 15.32 | 80.21 | 4.46 | 0.13 | 40.33 | 42.09 |
| 80 | 0.77 | -21.34 | 10.71 | 77.88 | 11.41 | 0.17 | 34.72 | 62.69 |
| 85 | 0.74 | -21.34 | 11.90 | 81.29 | 6.82 | 0.15 | 43.44 | 73.46 |
| 90 | 0.75 | -21.18 | 51.32 | 45.91 | 2.78 | 0.16 | 36.62 | 59.07 |
| 95 | 0.87 | N/A | 56.13 | 38.93 | 4.95 | 0.16 | 31.16 | 65.31 |
| 145 | 1.21 | -20.42 | 49.51 | 48.29 | 2.21 | 0.18 | 36.41 | 162.88 |
| 175 | 1.41 | -20.53 | 51.85 | 45.27 | 2.88 | 0.20 | 40.42 | 233.95 |
| 205 | N/A | N/A | N/A | N/A | N/A | N/A | N/A | 285.03 |
| 225 | N/A | -20.20 | 11.80 | 78.57 | 9.68 | N/A | 47.56 | 220.88 |
| 275 | 1.22 | -20.19 | 12.25 | 74.51 | 13.22 | 0.17 | 58.77 | 257.85 |
| 325 | 1.22 | -20.15 | 16.46 | 74.20 | 9.34 | 0.18 | 55.08 | 183.91 |
| 375 | 0.95 | -19.96 | 12.37 | 75.30 | 12.34 | 0.15 | 44.12 | N/A |
| 425 | 1.08 | -21.22 | 49.61 | 44.14 | 6.23 | 0.17 | 35.81 | 188.06 |
| 475 | 1.06 | -19.91 | 50.19 | 43.22 | 6.58 | 0.17 | 41.36 | 163.12 |
| 525 | 0.89 | -20.09 | 50.12 | 43.57 | 6.31 | 0.14 | 40.10 | 117.79 |
| 575 | 0.93 | -20.34 | 13.35 | 80.04 | 6.62 | 0.15 | 55.24 | 159.02 |
| 625 | 0.86 | -20.14 | 14.42 | 74.53 | 11.06 | 0.14 | 48.91 | 109.04 |
| 675 | 0.82 | N/A | 16.67 | 79.83 | 3.50 | 0.18 | 26.54 | 142.67 |
| 725 | 0.85 | -20.59 | 12.38 | 75.33 | 12.33 | 0.16 | 47.91 | 109.31 |
| 775 | 0.76 | -20.83 | 10.41 | 64.53 | 25.07 | 0.15 | 41.88 | N/A |
| 800 | 0.74 | -21.06 | N/A | N/A | N/A | 0.12 | 44.19 | 96.69 |

**Table S2**

| Depth (cm) | Replicate 1 | Replicate 2 | Replicate 3 | Mean | Maximum | Minimum | Confidence Intervals (95%) | |
| --- | --- | --- | --- | --- | --- | --- | --- | --- |
|  |  |  |  |  |  |  | **Upper** | **Lower** |
| 0 | 6.21 | 6.23 | 6.33 | 6.26 | 6.33 | 5.91 | 6.42 | 6.09 |
| 10 | 6.04 | 6.06 | N/A | 6.05 | 6.06 | 6.04 | 6.23 | 5.87 |
| 15 | 5.47 | 5.44 | 5.57 | 5.49 | 5.57 | 5.44 | 5.67 | 5.32 |
| 20 | 5.17 | 5.24 | 5.27 | 5.23 | 5.27 | 5.17 | 5.36 | 5.09 |
| 25 | 5.72 | 5.64 | 5.53 | 5.63 | 5.72 | 5.53 | 5.86 | 5.39 |
| 30 | 5.59 | 5.68 | 5.69 | 5.65 | 5.69 | 5.59 | 5.78 | 5.52 |
| 35 | 5.79 | 6.04 | 5.99 | 5.94 | 6.09 | 5.79 | 6.27 | 5.61 |
| 40 | 5.88 | 5.88 | 6.00 | 5.92 | 6.00 | 5.88 | 6.09 | 5.75 |
| 45 | 5.98 | 6.18 | 5.93 | 6.03 | 6.18 | 5.93 | 6.36 | 5.70 |
| 50 | 5.91 | 5.90 | 5.97 | 5.93 | 5.97 | 5.90 | 6.01 | 5.84 |
| 55 | 5.74 | 5.74 | 5.75 | 5.74 | 5.75 | 5.74 | 5.76 | 5.72 |
| 60 | 6.14 | 6.13 | 6.10 | 6.12 | 6.14 | 6.10 | 6.17 | 6.07 |
| 65 | 5.80 | 5.84 | 5.87 | 5.83 | 5.87 | 5.80 | 5.92 | 5.75 |
| 70 | 5.97 | 6.05 | N/A | 6.01 | 6.05 | 5.97 | 6.50 | 5.51 |
| 75 | 5.73 | 6.02 | 5.94 | 5.90 | 6.02 | 5.73 | 6.27 | 5.52 |
| 80 | 6.44 | 6.54 | 6.44 | 6.47 | 6.54 | 6.44 | 6.61 | 6.34 |
| 85 | 6.53 | 6.66 | 6.53 | 6.57 | 6.73 | 6.53 | 6.77 | 6.37 |
| 90 | 6.10 | 6.24 | 6.03 | 6.13 | 6.24 | 6.03 | 6.39 | 5.86 |
| 95 | 6.17 | 5.99 | 6.10 | 6.09 | 6.17 | 5.99 | 6.32 | 5.86 |
| 145 | 6.73 | 6.83 | 6.77 | 6.78 | 6.83 | 6.73 | 6.90 | 6.65 |
| 175 | 6.90 | 6.97 | 6.91 | 6.92 | 6.97 | 6.90 | 7.01 | 6.83 |
| 225 | 6.55 | 6.61 | 6.56 | 6.57 | 6.61 | 6.55 | 6.66 | 6.49 |
| 275 | 6.35 | 6.43 | 6.38 | 6.39 | 6.43 | 6.35 | 6.48 | 6.30 |
| 325 | 5.83 | 5.94 | 5.83 | 5.87 | 5.94 | 5.83 | 6.02 | 5.71 |
| 375 | 6.02 | 6.08 | 6.14 | 6.08 | 6.14 | 6.02 | 6.24 | 5.92 |
| 425 | 6.28 | 6.35 | N/A | 6.31 | 6.35 | 6.28 | 6.80 | 5.83 |
| 475 | 5.44 | 5.36 | 5.46 | 5.42 | 5.46 | 5.36 | 5.56 | 5.28 |
| 525 | 5.95 | 5.83 | N/A | 6.10 | 6.60 | 5.83 | 6.84 | 5.36 |
| 575 | 6.36 | 6.45 | 6.42 | 6.41 | 6.45 | 6.36 | 6.52 | 6.30 |
| 625 | 5.68 | 5.69 | 5.79 | 5.72 | 5.79 | 5.68 | 5.87 | 5.57 |
| 675 | 5.21 | 5.34 | N/A | 5.28 | 5.34 | 5.21 | 6.06 | 4.49 |
| 725 | 5.45 | 5.69 | 5.37 | 5.50 | 5.81 | 5.37 | 5.92 | 5.08 |
| 775 | 5.75 | 5.78 | N/A | 5.76 | 5.78 | 5.75 | 5.93 | 5.60 |
| 800 | 5.33 | 5.45 | 5.49 | 5.42 | 5.51 | 5.33 | 5.63 | 5.21 |
